# Extracellular vesicles as a potential source of tumor-derived DNA in advanced pancreatic cancer

**DOI:** 10.1371/journal.pone.0291623

**Published:** 2023-09-14

**Authors:** Morten Lapin, Kjersti Tjensvoll, Karoline Nedrebø, Eline Taksdal, Hans Janssen, Bjørnar Gilje, Oddmund Nordgård

**Affiliations:** 1 Department of Hematology and Oncology, Stavanger University Hospital, Stavanger, Norway; 2 Division of Biochemistry, The Netherlands Cancer Institute, Amsterdam, The Netherlands; 3 Department of Chemistry, Bioscience and Environmental Technology, Faculty of Science and Technology, University of Stavanger, Stavanger, Norway; Danmarks Tekniske Universitet, DENMARK

## Abstract

Tumor-derived extracellular vesicles (EVs) are reported to contain nucleic acids, including DNA. Several studies have highlighted the potential of EV-derived DNA (evDNA) as a circulating biomarker, even demonstrating that evDNA can outperform cell-free DNA (cfDNA) in terms of sensitivity. Here, we evaluated EVs as a potential source of tumor-derived DNA in patients with advanced pancreatic cancer. evDNA from both DNase-treated and untreated EV samples was analyzed to determine whether the DNA was primarily located internally or outside (surface-bound) the EVs. To assess whether methodology affected the results, we isolated EVs using four different methods for small EV isolation and differential centrifugation for isolating large EVs. Our results indicated that the DNA content of EVs was significantly less than the cfDNA content isolated from the same plasma volume (*p* < 0.001). Most of the detected evDNA was also located on the outside of the vesicles. Furthermore, the fraction of tumor-derived DNA in EVs was similar to that found in cfDNA. In conclusion, our results suggest that quantification of evDNA, as a source of tumor-derived DNA, does not add information to that obtained with cfDNA, at least not in patients with advanced pancreatic cancer.

## Introduction

Extracellular vesicles (EVs) are membrane-enclosed particles secreted by most cell types. They transfer molecules to other cells to influence recipient cell function and can vary in different features such as size, biogenesis, function, and cargo [1, 2]. EVs can be broadly categorized into three main classes based on size and the mode of biogenesis: a) exosomes, which are small (s)EVs (≤200 nm) shed by the fusion of multivesicular bodies to the plasma membrane; b) microvesicles or microparticles, which are large (l)EVs (>200 nm) shed by outward budding of the plasma membrane; and c) apoptotic bodies, which are released from fragments of apoptotic cells [1–5].

Subgroups of EVs are reported to contain nucleic acids, and the discovery of DNA inside tumor-derived EVs has spawned numerous publications investigating its potential as a biomarker in cancer [6–12]. Recent reports also have implied that the tumor fraction of EV-derived DNA (evDNA) might be higher than the tumor fraction in comparative cell-free DNA (cfDNA) in patients with cancer, making EVs a potential source of tumor-derived DNA in the circulation [13–15]. However, several studies have reported conflicting results [16, 17]. It has been proposed that evDNA is bound outside the vesicles, with only a minute portion present inside [16]. Another hypothesis is that DNA is not associated with circulating EVs but rather is carried by multivesicular endosomes and released into the extracellular space upon fusion with the plasma membrane [17]. Further studies are needed to clarify whether EVs actually contain DNA, and if so, the biomarker potential of such DNA.

Ultracentrifugation has long been considered the preferred method for sEV isolation [18]. However, this method co-isolates high-density lipoproteins [19], is quite cumbersome, may result in clumping of EVs, and can lead to damage of EVs and sample loss [20]. Other isolation methods include size exclusion chromatography, density gradient centrifugation, filtration, and polymer-based precipitation [20]. In recent comparison studies, several of these methods have outperformed ultracentrifugation in terms of sEV recovery [21–24]. The focus of these comparison studies has been on sEV isolation efficacy, purity, and the impact of isolation methods on downstream RNA and miRNA analyses. To the extent that evDNA exists, how specific isolation methods affects yield and how different methods might isolate distinct EV populations with varying tumor-derived DNA content has yet to be examined.

In this study, we evaluated whether EVs are a potential source of tumor-derived DNA in patients with advanced pancreatic cancer. Overall evDNA concentration and tumor-derived (mutated) DNA fraction were determined in both DNase-treated and untreated EVs isolated from plasma samples. To also determine the effects of different isolation methods on results, we isolated evDNA using four methods for sEVs and differential centrifugation for isolating lEVs.

## Results

### EV populations isolated by different methods differ in size, purity, and content

**Fig 1:**
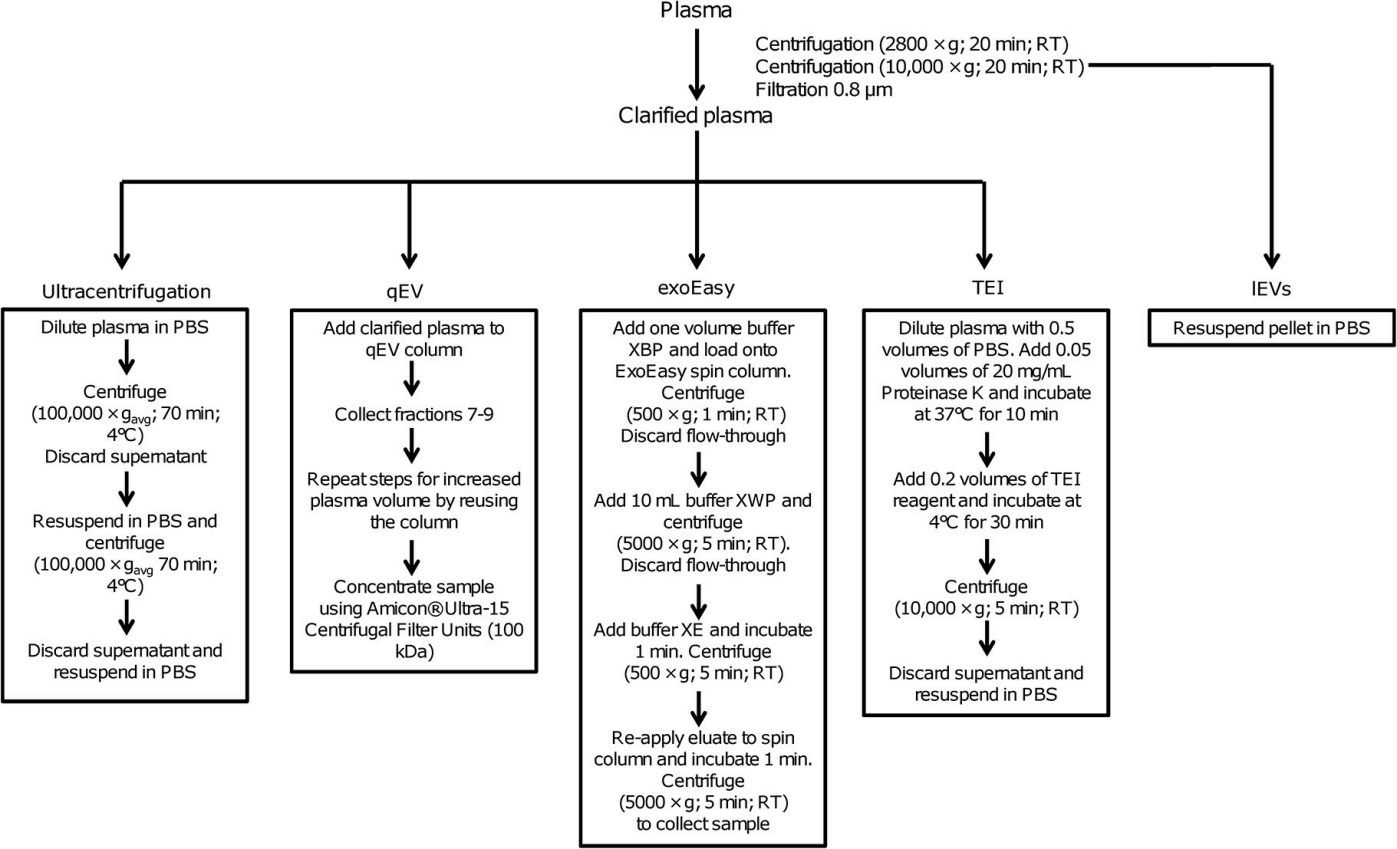
Schematic overview of methods used for isolating EVs. Plasma, clarified by differential centrifugation and filtration using a 0.8-μm membrane filter, served as input for each isolation method. lEVs were co-isolated after differential centrifugation of plasma and before plasma filtration. TEI: total exosome isolation; lEVs: large extracellular vesicles.

EVs were isolated from clarified healthy volunteer plasma using four sEV isolation methods: ultracentrifugation, qEV size exclusion chromatography, exoEasy membrane affinity spin columns, and Total Exosome Isolation (TEI) precipitation. In addition, lEVs were isolated by differential centrifugation. The isolation methods are summarized in **Fig 1**.

**Fig 2:**
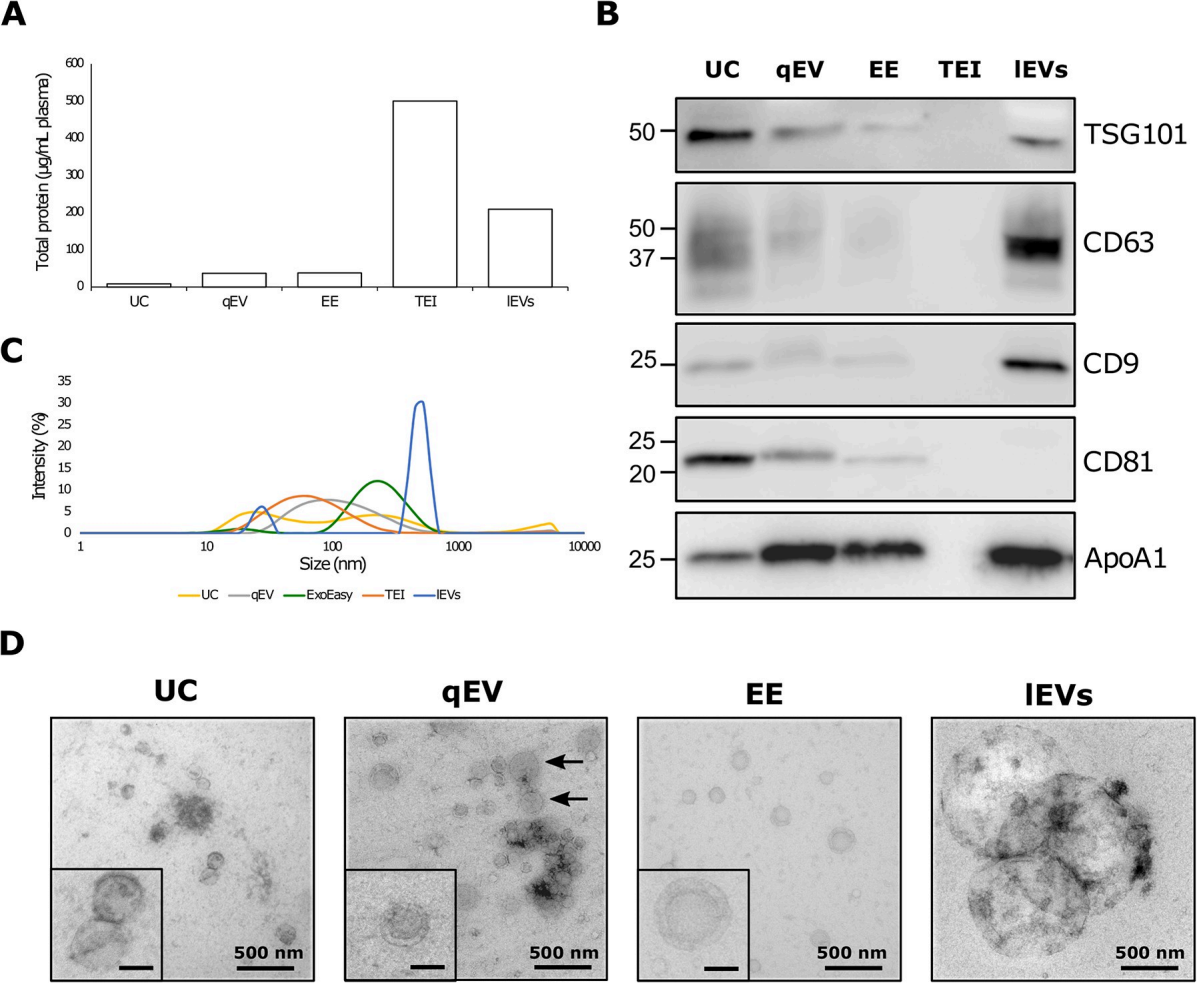
Characterization of EVs isolated from plasma using different isolation methods. A) Relative protein level per milliliter of plasma in EV isolates, normalized to UC. B) Western blot analysis of EV protein markers (TSG101, CD63, CD9, CD81) and the contamination marker ApoA1 in 10 μg EV proteins. C) Particle size distributions measured by DLS intensity. D) TEM images of EVs counterstained with uranyl acetate; scale bars in inset images = 100 nm, arrows indicate chylomicrons. UC: ultracentrifugation; EE: exoEasy; TEI: total exosome isolation; lEVs: large extracellular vesicles. The western blot data is composed of images from multiple membranes. Adjustment of brightness and contrasts of entire images was performed to obtain similar background staining of membranes. Full, unadjusted and uncropped images can be found in the supplemental materials (**S1 Raw images**).

To characterize the EV populations isolated with the different methods, we first determined total protein levels and content of the exosome protein markers TSG101, CD63, CD9, and CD81 and the contamination marker ApoA1 [5] with western blot (WB) analyses. The total protein yield was lowest for EVs isolated using ultracentrifugation. Compared with ultracentrifugation, total protein levels were 4-fold higher in EVs isolated with qEV and exoEasy, 25-fold higher in lEVs, and 60-fold higher in EVs isolated with TEI (which co-precipitate plasma proteins; **Fig 2A**). In contrast, WB analyses demonstrated that the content of all exosome markers was elevated in EVs isolated with ultracentrifugation compared with those isolated by qEV and exoEasy, indicating higher EV purity (**Fig 2B**). lEVs, on the other hand, had high levels of the exosome markers TSG101, CD63, and CD9, but not CD81, demonstrating a difference in EV phenotypes between lEV and sEVs. Of note, no signal for exosome protein markers was observed in TEI protein extractions. The contamination marker ApoA1 was detected at varying levels in all EV samples except TEI and was lowest with ultracentrifugation and quite prominent for qEV (which co-isolates ApoA1-containing chylomicrons) and lEVs.

The EV populations were further characterized using dynamic light scattering (DLS) and transmission electron microscopy (TEM). The size distribution of sample particles as determined by DLS demonstrated size differences among the EV populations, although all populations were non-uniform and contained particles of the expected size (≤200 nm for sEVs, >200 nm for lEVs; **Fig 2C**). The particles isolated with ultracentrifugation and exoEasy fell into two distinct groups. The larger size population was consistent with sEVs, whereas the smaller population corresponded to the size of high-density lipoproteins, which are co-isolated by ultracentrifugation [19]. Particles isolated with qEV and TEI, in contrast, fell into single, broader groups. The lEV particles also fell into two distinct groups, but contained larger particles than did the sEV populations (**Fig 2C**).

TEM was performed for EV samples isolated by ultracentrifugation, qEV, and exoEasy, and for lEVs (**Fig 2D**). The TEI sample was excluded from these analyses because of low vesicle purity. All investigated samples produced typical cup-shaped EVs (**Fig 2D**); the ultracentrifugation, qEV, and exoEasy samples all contained EVs of 80–200 nm and with varying degrees of contaminants, whereas the lEV sample contained larger vesicles up to 1 μm in size. In the exoEasy sample, we also observed some strange particles that were suspected to have originated from the column (**S1 Fig**).

### DNA measurements and mutant *KRAS* detection in plasma EVs isolated with different methods

**Fig 3:**
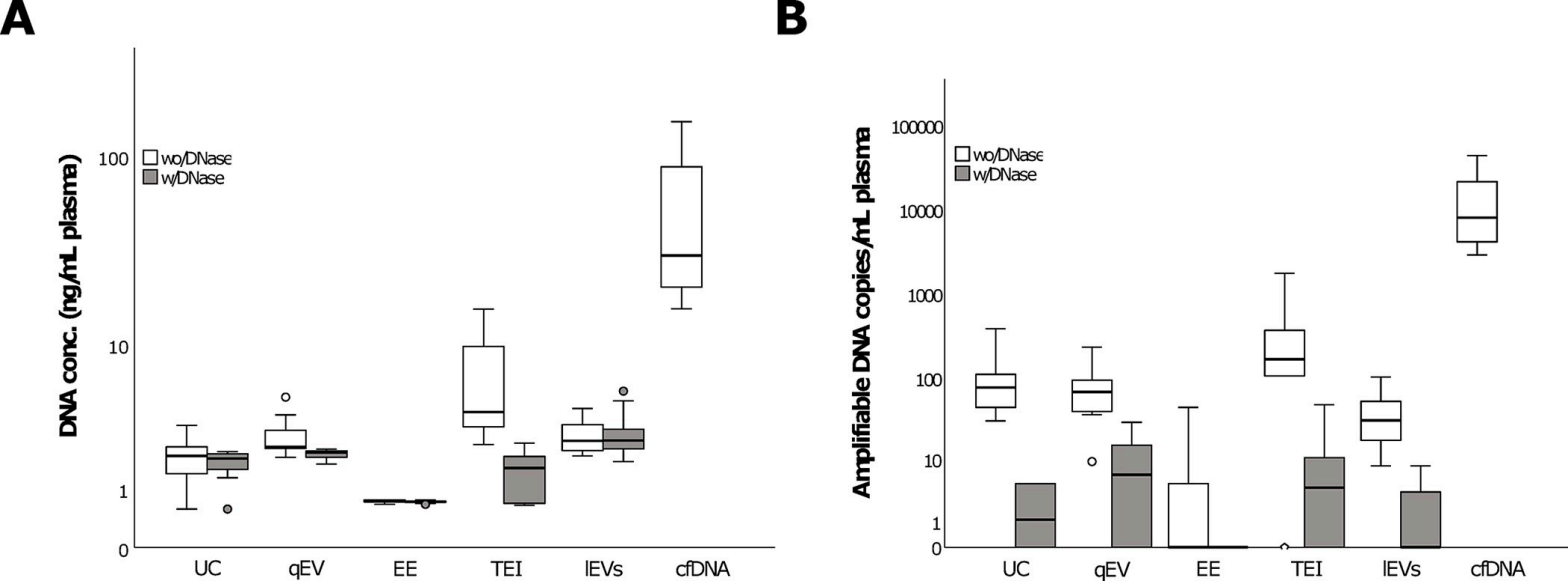
Comparison of cfDNA and evDNA levels in vesicles isolated using different EV isolation methods. A) DNA concentration in EVs, with and without treatment with DNase, compared to cfDNA, measured by fluorometric quantification. B) Amplifiable *KRAS* DNA copies in EVs, with and without treatment with DNase, compared to cfDNA, measured by ddPCR. UC: ultracentrifugation; EE: exoEasy; TEI: total exosome isolation; lEVs: large extracellular vesicles.

EVs were isolated from 10 plasma samples from patients with advanced pancreatic cancer, using all four sEV isolation methods and lEV isolation. Although we were not able to detect EV protein markers in the TEI sample we decided to also include this method as it is a commercial method marketed as an extracellular vesicle isolation method. To investigate whether EVs contain DNA, we sought to isolate DNA from all EV samples and isolated cfDNA from the same patient samples for comparison. In addition, to investigate if potential evDNA was primarily located inside EVs or was bound to the EV surface, we either DNase-treated the EVs to degrade surface-bound DNA or left them untreated. In samples not treated with DNAse, we measured relatively low concentrations of evDNA by fluorometric quantification (**Fig 3A**). However, EVs isolated using TEI contained almost twice the evDNA of those isolated with the other methods, and EVs isolated with exoEasy barely contained detectable DNA (in 2/10 samples). In DNase-treated samples, EVs isolated using TEI had 60% reductions in evDNA concentration (**Fig 3A**), indicating that the DNA isolated with this method was primarily located on the outside of the vesicles or in other particles in the samples. In contrast, only a small reduction in evDNA concentration (<10%) was observed for EVs isolated by qEV, ultracentrifugation, and lEVs. Compared with the cfDNA concentration, the concentration of evDNA in EV samples was very low (>10-fold lower; *p* < 0.001 for all comparisons), whether samples were DNAse treated or not (**Fig 3A**).

The evDNA from all EV samples and cfDNA from the same plasma volume were subjected to *KRAS* mutation detection using ddPCR (amplicon length = 57 bases), which allowed us to determine the number and concentration of amplifiable *KRAS* copies (both wild-type and mutant DNA copies; **Fig 3B**). Among those not treated with DNase, TEI samples had the highest concentration of amplifiable DNA copies, whereas almost no DNA was detected in exoEasy samples, similar to measurements of DNA using fluorometric quantification (**Fig 3**). However, in contrast to the amount of DNA identified by fluorometric quantification, only minute amounts of amplifiable DNA were detectable by ddPCR after DNase treatment in all EV samples, suggesting that the EVs carry little or no amplifiable DNA internally. In comparison to evDNA, the concentration of amplifiable cfDNA copies detected by ddPCR was significantly higher (*p* < 0.001 for all comparisons): >50-fold higher than untreated EVs and >1000-fold higher than DNase-treated EVs (**Fig 3B**). Furthermore, the concentration of amplifiable cfDNA copies was comparable to the concentration measured using the Qubit fluorometer (median 27.13 vs 30.26 ng/mL plasma, *p* = 0.436), in contrast to evDNA concentration, which was significantly decreased (*p* < 0.05 for all EV isolation methods, both with and without DNase treatment).

Variant allele frequencies (VAFs) of *KRAS* mutations determined by ddPCR, reflecting tumor-derived DNA content, were compared among evDNA samples and cfDNA samples. The cfDNA samples all contained detectable point mutations in *KRAS* codon 12, with VAFs ranging from 0.19% to 47.1% (**Table 1)**. In contrast, *KRAS* mutations were detected in only 4/10 untreated evDNA samples (using any EV isolation method), with VAFs ranging from 2.3% to 59%, and in none of the DNase-treated samples (**Table 1**). In fact, many of the DNase-treated samples did not contain amplifiable *KRAS* DNA at all. The VAFs detected in the untreated EV samples were comparable to those in cfDNA in corresponding samples.

**Table 1:** Mutated *KRAS* VAF (%) detected in evDNA and cfDNA.

	UC		qEV		EE		TEI		lEVs		cfDNA
Sample ID	untreated	DNase	untreated	DNase	untreated	DNase	untreated	DNase	untreated	DNase	untreated
1	0	-	0	0	-	-	-	-	0	-	4.8
2	25	-	0	-	0	-	0	0	0	-	4.9
3	0	0	0	0	-	-	-	0	0	0	2.07
4	0	0	0	0	0	-	0	0	0	-	0.26
5	40	0	38	0	18	-	47.6	-	59	0	47.1
6	0	0	0	0	-	-	0	-	0	-	0.19
7	0	-	15	-	-	-	4.1	0	0	0	9
8	0	-	0	-	0	-	0	0	0	-	6.3
9	0	0	0	0	-	-	0	0	0	-	0.39
10	0	0	0	0	-	-	2.3	0	0	-	2.9

(-) indicates samples without amplifiable DNA

DNase: DNase-treated; UC: ultracentrifugation, EE: exoEasy; TEI: total exosome isolation; lEVs: large extracellular vesicles

## Discussion

In this study, we sought to investigate whether evDNA could be a potential source of tumor-derived DNA in patients with advanced pancreatic cancer, and whether the isolation method used affects evDNA content. Previous findings have shown that different EV isolation methods isolate EV populations with different purity, nucleic acid content, and protein content [21–24]. In line with these earlier reports, we also found that different isolation methods isolate EV populations with different purity and protein content. In addition, our findings demonstrate disparities in evDNA content among EVs isolated with different methods. For instance, although most EV populations contained similar but minute amounts of evDNA, EVs isolated by affinity purification (exoEasy) contained almost no DNA. Furthermore, our findings also suggest that a large portion of the evDNA was located on the outside of the vesicles, as DNAse treatment of the EVs led to decreasing DNA content. Moreover, the evDNA contained few amplifiable DNA copies, independent of the isolation method. Consequently, only a few patient samples contained tumor-derived mutant DNA that could be detected using ddPCR, and these samples corresponded to samples with high VAF detected in cfDNA. The VAFs detected in evDNA also were quite similar to the VAFs detected in cfDNA, suggesting a limited benefit of adding evDNA analysis or using it as an alternative to cfDNA.

The data presented here contrast with findings from several previous studies that reported relatively high levels of evDNA and frequent detection of genetic aberrations in EVs from patients with cancer [9, 11, 13, 15]. A possible explanation for the discrepancy is that these studies have used DNase I for DNA digestion in PBS buffer, which has a too-high salt concentration for efficient digestion (according to the manufacturers). Furthermore, whether these studies relied on DNase-treated vesicle pellets or vesicle suspensions is unclear. It is possible that there was co-isolation of cfDNA protected from nuclease degradation or evDNA located on the outside of the vesicles, which seems to be the predominant source of evDNA [16]. However, these factors only partly explain the discrepancy, as our results for EVs that were not treated with DNase also indicated relatively low concentrations of evDNA. Another possible factor in the discrepancy could have been selection of patients with high mutational burden in previous studies and large plasma volumes (up to 12 mL) used to isolate evDNA [15], which would make mutations in evDNA easier to detect. In line with our results, however, are recent studies suggesting that only a small percentage (<1%) of sEVs from patients with cancer contain DNA [25], and that DNA is rarely associated with EVs [26]. Recently, data from a seminal study also showed that exosomes, the EVs thought to carry DNA, do not contain any DNA at all [17].

We found it surprising that DNA detected in EVs in our study was not easily amplifiable, as previous results have indicated that evDNA from both sEVs and lEVs is primarily high molecular weight DNA [12, 16, 27]. A possible explanation for the low amount of amplifiable *KRAS* copies in our EV samples could be that the isolated evDNA was enriched for certain parts of the genome only, although this inference finds little support in previous research demonstrating that evDNA spans the whole genome [16, 27]. A more likely explanation is that the isolated DNA consisted of smaller DNA fragments/degraded DNA that proved difficult to amplify.

Both sEVs and lEVs are reported to contain high levels of single-stranded (ss)DNA [12, 16], but earlier studies relied on Qubit fluorometry for DNA quantification and did not consider the rather large spillover from double-stranded DNA when measuring ssDNA with this method. We made initial attempts to estimate ssDNA concentration in evDNA using the Qubit fluorometer, but our results indicated that most of the ssDNA in EVs measured in this way was in fact double-stranded DNA, in line with the documentation of the Qubit™ ssDNA Assay Kit. Other detection methods more specific to ssDNA should be used in the future to estimate ssDNA concentration.

This study has several limitations. First, we used only 1 mL plasma for isolation of vesicles and did not use whole samples for ddPCR analyses. This choice is likely to have affected the sensitivity of the *KRAS* mutation detection. Second, patient plasma samples were frozen at -80°C before EV isolation, which could have affected vesicle number and integrity. However, previous data have demonstrated that one freeze–thaw cycle at -80°C has only modest effects [28]. Third, we isolated EVs from patients with pancreatic cancer and cannot exclude that other cancer types release higher numbers of EVs or EVs containing higher levels of evDNA. Finally, we used a method for determining EV size that is not ideal for samples containing particles of different sizes. Thus, the reported size distributions might differ from those of previous studies using more quantitative methods.

In conclusion, our results indicate that EVs contain only minute amounts of DNA. Most of the detected evDNA, at least the amplifiable DNA, also was located on the outside of vesicles. Obtaining evDNA concentrations appropriate for downstream applications would require isolation of EVs from a much higher volume of plasma (>10 mL) than is usually collected. Furthermore, the VAFs of tumor-derived DNA in EVs were similar to the tumor fraction found in cfDNA, and thus do not seem to add further information as a source of circulating tumor-derived DNA.

## Materials and methods

### Study population

The patient samples in this study (n = 10, collected from seven patients between April 2019 and February 2020; detailed in **S1 Table**) were selected from a cohort with advanced pancreatic cancer prospectively recruited to an observational study at Stavanger University Hospital. The included samples were accessed between April and August 2020, and chosen based on the presence of cfDNA mutations in the *KRAS* gene previously determined in aliquots of the same plasma samples. We also included samples from a healthy control for EV population characterization. All patients and the healthy control provided written informed consent to participate in the study. The project was conducted in accordance with the Declaration of Helsinki and approved by the Regional Committee for Medical and Health Research Ethics (REK-Vest 2015/2011).

### Clarified plasma preparation

Peripheral venous blood (9 mL in EDTA tubes) was first centrifuged at 2000 × *g* for 10 min at room temperature (RT) and the plasma stored at −80°C or processed directly. To produce clarified plasma, we performed sequential centrifugations at 2800 × *g* for 10 min and at 10,000 × *g* for 20 min at RT to eliminate cells, larger vesicles, and debris, followed by filtering through a 0.8-μm membrane filter (Millex-AA Syringe Filter Unit, 0.80 μm, Merck). For the control sample, 2–10 mL plasma was processed depending on EV isolation method. For the patient samples, 4 mL plasma was processed, 1 mL for each EV isolation method.

### Isolation of large EVs

After the centrifugation at 10,000 × *g* in the clarified plasma preparation, the pellet containing lEVs (from 1 mL plasma) was resuspended in filtered phosphate-buffered saline (PBS; Millex-MP Syringe Filter Unit, 0.22 μm, Merck) and subjected directly to downstream analyses.

### Ultracentrifugation

Clarified plasma was transferred to 4 mL (38 mL for larger plasma volumes) Thickwall Polycarbonate Tubes (Beckman Coulter®), and filtered PBS was added to a final volume of 3.8 mL (16 mL for larger plasma volumes). Next, the samples were centrifuged in a Beckman Coulter Optima™ XPN-100 ultracentrifuge at 100,000 × *g*_avg_ for 70 min at 4°C using a Type 70 Ti Fixed-Angle rotor (k-factor = 186.1) to pellet sEVs. The pellet was resuspended to the initial volume in filtered PBS as a wash and centrifuged a second time at 100,000 × *g*_avg_ for 70 min at 4°C. The final pellet was resuspended in filtered PBS and subjected directly to downstream analyses.

### Size exclusion chromatography

Size exclusion chromatography was performed using qEV original columns (for DNA-isolation; Izon Science) or qEV2 70 nm columns (for WB and TEM; Izon Science) according to the manufacturer’s instructions. After equilibration of the column, the recommended volume of clarified plasma was loaded onto the qEV columns and fractions 7–9 were eluted. The process was repeated for increased plasma volumes, with a column-washing step in between. Eluted fractions (containing sEVs) were combined and concentrated using Amicon® Ultra-15 Centrifugal Filter Units (100 kDa, Merck) and subjected directly to downstream analyses.

### exoEasy membrane affinity spin columns

Isolation of sEVs using membrane affinity spin columns was performed according to the manufacturer’s instructions (exoEasy Maxi Kit, Qiagen). Briefly, one volume of XBP buffer (Qiagen) was added to clarified plasma and the sample then added to a spin column and centrifuged at 500 × *g* for 1 min. Next, 10 mL XWP buffer (Qiagen) was added to the spin column and the sample centrifuged at 5000 × *g* for 5 min. Finally, the sEVs were eluted in 400 μL buffer XE (Qiagen) by sample centrifugation at 500 × *g* for 5 min, re-application of the eluate to the spin column, and centrifugation at 5000 × *g* for 5 min. When isolating sEVs from more than 4 mL of clarified plasma (for WB and TEM), we combined multiple eluates and concentrated them to a final volume of 400 μL using Amicon® Ultra-15 Centrifugal Filter Units (100 kDa, Merck). The final eluate was collected and subjected directly to downstream analyses.

### Total exosome isolation precipitation

Precipitation of sEVs was performed with the Total Exosome Isolation Reagent (from plasma; Thermo Fisher Scientific), according to the manufacturer’s instructions. Briefly, clarified plasma was diluted with 0.5 volumes of filtered PBS, 0.05 volumes of 20 mg/mL Proteinase K (Invitrogen™) was added and the sample was incubated at 37°C for 10 min to remove plasma proteins, before being mixed with 0.2 volumes of precipitation reagent. The sample was then incubated at 4°C for 30 min and centrifuged at 10,000 × *g* for 5 min at RT. The resulting pellet was resuspended in filtered PBS and subjected directly to downstream analyses.

### Protein concentration determination

EV samples were lysed by the addition of 0.25 volumes of ice-cold 5× RIPA buffer (50 mM Tris-HCl pH 7.4, 750 mM NaCl, 0.5% sodium deoxycholate, 0.5% SDS, 5% Triton) with protease inhibitor cocktail (Sigma-Aldrich) and incubation for at least 5 min on ice. Total protein concentration was measured using the Pierce™ BCA Protein Assay Kit (Thermo Fisher Scientific) according to the manufacturer’s instructions. Briefly, 200 μL working reagent (50 parts reagent A to 1 part reagent B) was added to a 25-μL EV sample in a microplate well and the microplate shaken at low speed for 30 s. Next, the microplate was incubated at 37°C for 30 min before absorbance was read at 562 nm on a Bio-Rad 680 XR plate reader. Protein concentration was calculated using a standard curve of bovine serum albumin at known concentrations.

### Western blot analysis of exosome protein markers

EV samples were lysed in 5× RIPA buffer as described above. Proteins were denatured by the addition of 0.33 volumes of 4× Laemmli loading buffer (750 mM Tris-HCl pH 6.8, 5% SDS, 40% glycerol, 80 mM DTT) with or without (for CD9 and CD63 detection) DTT and incubated for 10 min at 70°C. EV proteins (10 μg total protein per lane) were separated according to size using 4%–20% Mini-PROTEAN® TGX™ Precast Protein Gel (Bio-Rad) and a Tris-Glycine buffer system. The proteins were blotted onto polyvinylidene fluoride membranes (Bio-Rad) in a semi-dry blotting system for 45 min at 15 V (max current 300 mA), with membrane wetting and positive electrode filter paper in Plus buffer (25 mM Tris base, 20% methanol) and the negative electrode filter paper in Minus buffer (25 mM Tris base, 40 mM 6-aminohexanoic acid [Sigma-Aldrich], 20% methanol). We then incubated the membranes in Antigen Pretreatment Solution (SuperSignal Western Blot Enhancer kit, Sigma-Aldrich) for 10 min at RT with shaking (this and all subsequent incubation steps were done with shaking). The membranes were then rinsed 5 times in ultrapure water and blocked by incubation for 30 min in TBST buffer (10 mM Tris-HCl, pH 8.0, 150 mM NaCl, 0.05% Tween 20) with 5% skim milk. Membranes next were rinsed three times in TBST buffer, washed for 5 min in TBST buffer, and incubated for 2 h at RT with primary antibody in Primary Antibody Diluent (SuperSignal Western Blot Enhancer kit, Sigma-Aldrich). The following primary antibodies and concentrations were used: anti-TSG101 (polyclonal, Abcam ab30871, dilution 1:1000), anti-CD63 (TS63, Thermo Fisher Scientific 10628D, dilution 1:1000), anti-CD9 (SN4 C3-3A2, Thermo Fisher Scientific 14-0098-82, dilution 1:1000), anti-CD81 (polyclonal, Thermo Fisher Scientific PA579004, dilution 1:1000), and anti-ApoA1 (EP1368Y, Abcam ab52945, dilution 1:1000). The membranes were then washed 3 × 5 min and 1 × 15 min in TBST with 5% skim milk. Incubation with secondary antibodies was done for 1 h at RT, using horseradish peroxidase–conjugated anti-mouse IgG (Thermo Fisher Scientific A28177, dilution 1:10000) or anti-rabbit IgG (Thermo Fisher Scientific 31464, dilution 1:10000), depending on the primary antibody. The membranes were then rinsed three times and washed 4 × 5 min with TBST buffer. Chemiluminescent detection of secondary antibodies was performed with the SuperSignal™ West Femto Maximum Sensitivity Substrate (Thermo Fisher Scientific) and the Azure 300 scanner (Azure Biosystems), according to the instructions of the manufacturer. All chemiluminescense images were processed with the GIMP image processing software (version 2.10), including moderate adjustment of brightness and contrasts of entire images, to obtain similar background staining of membranes. Full, unadjusted, images can be found in the supplemental materials (**S1 Raw images**).

### Analysis of particle size

The size distribution of particles in the EV samples was analyzed by dynamic light scattering using a Zetasizer Nano ZS (Malvern Panalytical) instrument, according to the manufacturer’s instructions. A low-volume quartz batch cuvette (ZEN2112) was loaded with 50 μL EV samples and analyzed in triplicate according to instructions. Scattered light intensity distributions were used to determine particle sizes. Carboxylated polystyrene particles (CPC100, CPC200, Izon Science) were used as size controls.

### Transmission electron microscopy

Unfixed EVs were loaded onto formvar/carbon-coated copper grids (3.05 mm, 300 mesh, TAAB Laboratories Equipment Ltd) and stained with uranyl acetate. The grids were incubated with the EVs for 20 min, washed three times in filtered ultrapure water, and incubated for 5 min in 0.3% uranyl acetate/2% methylcellulose at RT. Excess uranyl acetate/methylcellulose staining was removed from the grids by touching them to a filter paper (Whatman #2) at a 45° angle. The grids were dried overnight at RT and investigated for the presence of EVs using a Tecnai12G2 electron microscope fitted with a Veleta camera (Thermo Fisher, Eindhoven, the Netherlands) at 120 kV.

### DNase treatment

Prior to isolation of evDNA, half of the EV samples were DNase-treated to digest DNA located outside of the vesicles. The DNase treatment was performed using TURBO™ DNase (Invitrogen) by the addition of 2 U TURBO™ DNase and 20 μL 10× TURBO™ DNase reaction buffer to 175 μL of the EV sample, followed by incubation of the sample in a water bath at 37°C for 30 min. Inactivation of the DNase was performed in the first step of the DNA isolation protocol by the addition of proteinase K and lysis buffer AL (containing guanidine hydrochloride), directly after DNAse treatment.

### DNA isolation

Isolation of evDNA from DNase-treated and untreated EV samples was performed using the DNeasy Blood & Tissue Kit (Qiagen) according to the manufacturer’s instructions for cultured cells. Briefly, DNase-treated EV samples were diluted to 200 μL in PBS before isolation, and the DNA then eluted in 50 μL buffer AE (Qiagen). cfDNA was isolated from 1 mL of plasma using the QIAamp Circulating Nucleic Acid kit (Qiagen), as described by the manufacturer. cfDNA was eluted in 50 μL of Buffer AVE (Qiagen). All DNA samples were stored at –80°C until further analysis.

### Determination of DNA concentration

DNA concentration was measured using the Qubit™ dsDNA HS assay kit (Thermo Fisher Scientific) according to the manufacturer’s instructions. For measurement, we used 10 μL evDNA or 1 μL cfDNA input on a Qubit 2.0 Fluorometer (Thermo Fisher Scientific).

### ddPCR

*KRAS* mutations in codons 12/13 in patient samples were determined using the ddPCR™ *KRAS* G12/G13 Screening Kit (Bio-Rad), which screens for the seven most common mutations in the *KRAS* gene on a QX200 Droplet Digital PCR system (Bio-Rad), according to the manufacturer’s instructions. Samples were run in duplicate in 20 μL reactions (10 μL 2× ddPCR Supermix for Probes, 1 μL 20× target and wild-type primers/probes, 0.5 μL MseI restriction enzyme, and 8.5 μL sample) using appropriate controls, producing a median 31364 (Interquartile range, 29302–33642) droplets per sample.

### Statistical analyses

All statistical analyses were performed with IBM SPSS version 26.0 (www.spss.com). All tests were two-sided, and *p* values less than 0.05 were considered statistically significant. Statistical analyses were performed using the Student’s t-test, or the Mann–Whitney U test for non-parametric data.

### Data reporting

EV isolation and characterization in this study were performed and reported according to the MISEV2018 guidelines [5] and registered in the EV-TRACK database (EV-TRACK ID: EV220369) [29].

## Supporting information

S1 Fig TEM images of EVs isolated by ExoEasy affinity purification and stained by uranyl acetate. (PDF)

S1 Raw images Raw images of western blot analyses visualized in white light and by chemiluminescence. (PDF)

S1 Table Patient characteristics and sample information. (XLSX)
