# Supplementary material for: Extracellular vesicles as a potential source of tumor-derived DNA in advanced pancreatic cancer
Source: PLoS One. 2023 Sep 14;18(9):e0291623. doi: 10.1371/journal.pone.0291623 (PMC10501680; doi:10.1371/journal.pone.0291623)
Supplement: S1 Fig — (PDF) [file pone.0291623.s001.pdf]

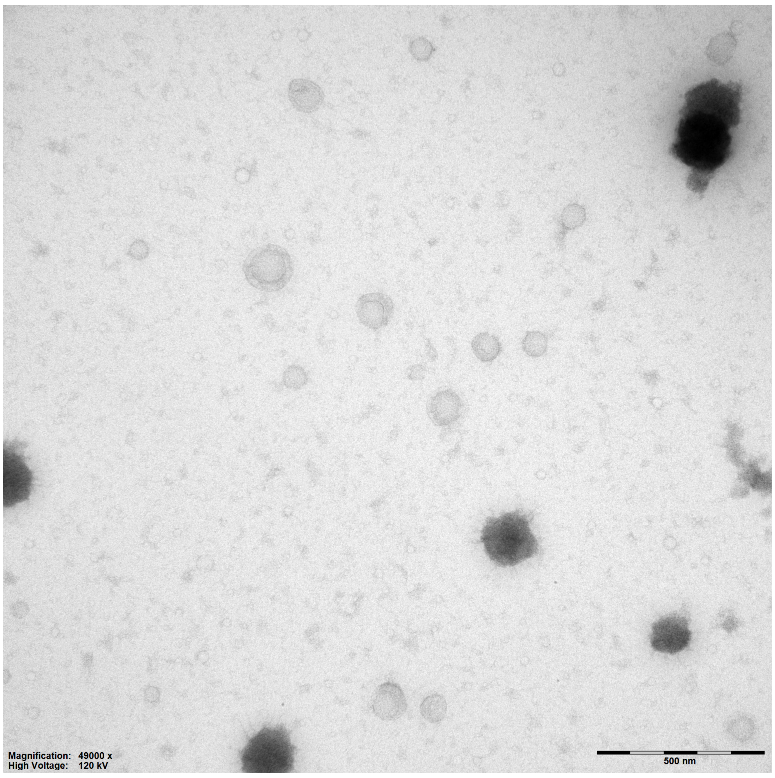

Supplemental figure S1: TEM images of EVs isolated by ExoEasy affinity purification and stained by uranyl acetate; scale bar in inset image = 100 nm. The entire image is shown, including some dark particles that seem to originate from the columns.
