# Supplementary material for: Extracellular vesicles as a potential source of tumor-derived DNA in advanced pancreatic cancer
Source: PLoS One. 2023 Sep 14;18(9):e0291623. doi: 10.1371/journal.pone.0291623 (PMC10501680; doi:10.1371/journal.pone.0291623)
Supplement: S1 Raw images — (PDF) [file pone.0291623.s002.pdf]

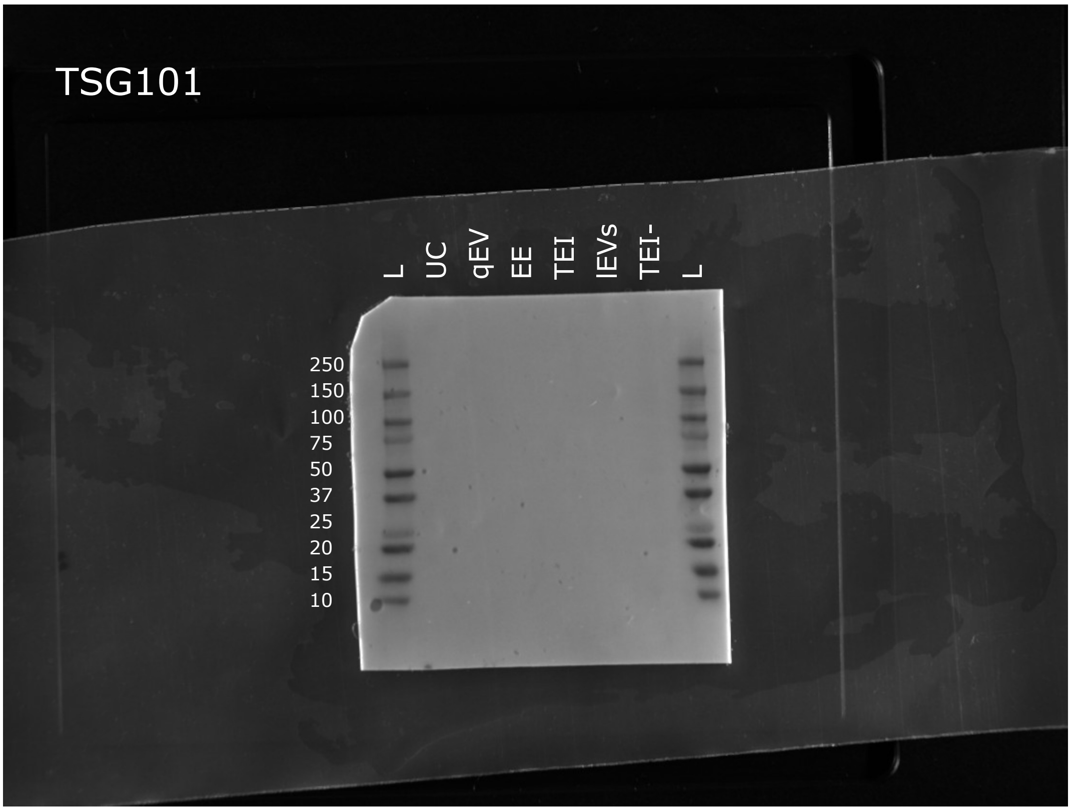

Supplemental figure S2: Uncropped, unadjusted western blot analysis of TSG101 in 10 µg vesicle proteins isolated by ultracentrifugation (UC), size-exclusion chromatography (qEV), ExoEasy affinity purification (EE), Total exosome isolation kit with (TEI) and without (TEI-) protease treatment, and large vesicle sedimentation (lEVs). The image is visualized in white light. Ladder (L) sizes are added for reference.

TSG101

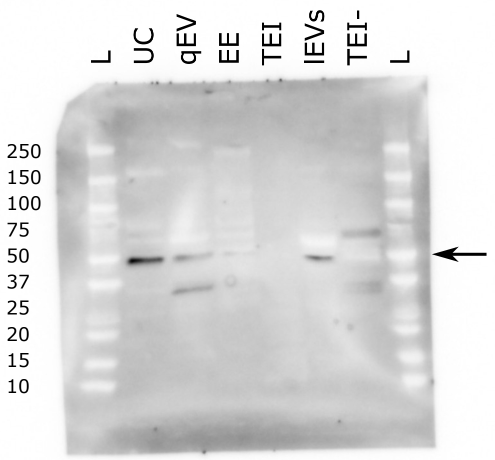

Supplemental figure S3: Uncropped, unadjusted western blot analysis of TSG101 in 10 µg vesicle proteins isolated by ultracentrifugation (UC), size-exclusion chromatography (qEV), ExoEasy affinity purification (EE), Total exosome isolation kit with (TEI) and without (TEI-) protease treatment, and large vesicle sedimentation (lEVs). The image is visualized by chemiluminescence. Ladder (L) sizes are added for reference. The contrast is set automatically by the Azure 300 scanner. Arrow indicates the expected size of the TSG101 protein.

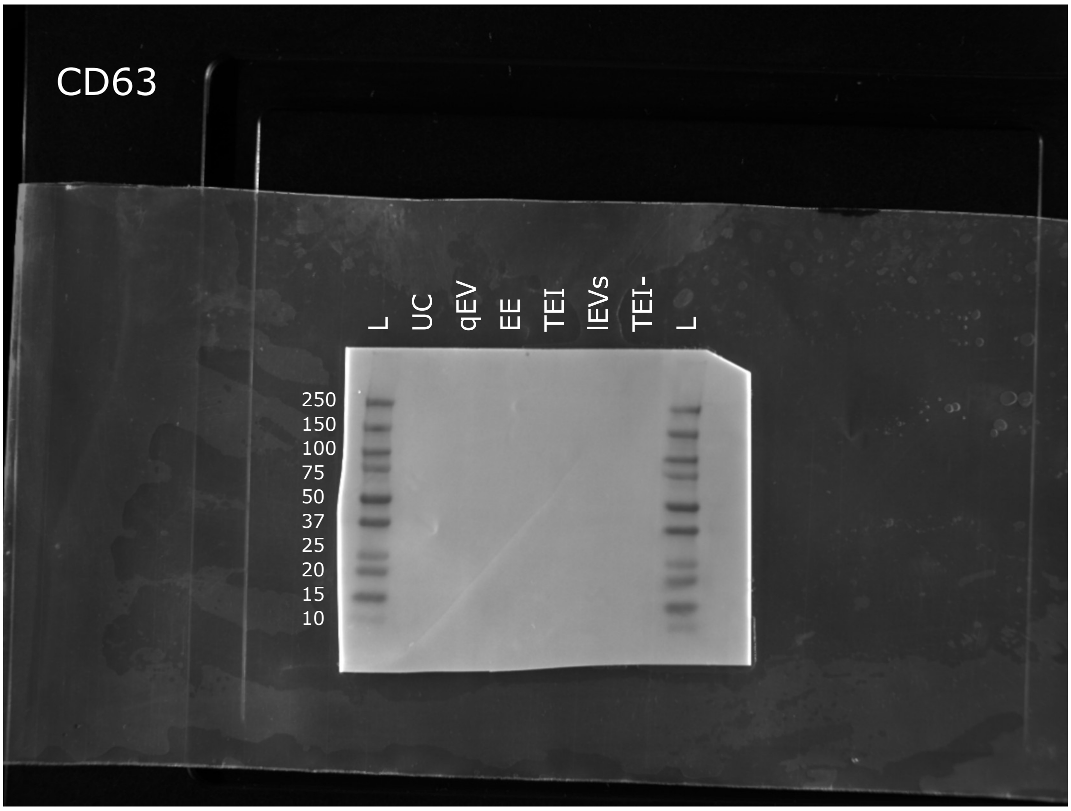

Supplemental figure S4: Uncropped, unadjusted western blot analysis of CD63 in 10 µg vesicle proteins isolated by ultracentrifugation (UC), size-exclusion chromatography (qEV), ExoEasy affinity purification (EE), Total exosome isolation kit with (TEI) and without (TEI-) protease treatment, and large vesicle sedimentation (lEVs). The image is visualized in white light. Ladder (L) sizes are added for reference.

CD63

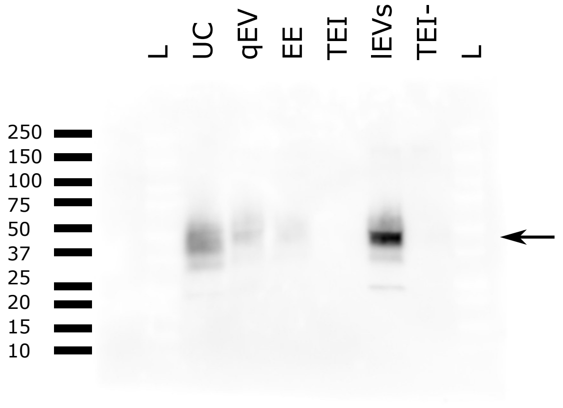

Supplemental figure S5: Uncropped, unadjusted western blot analysis of CD63 in 10 µg vesicle proteins isolated by ultracentrifugation (UC), size-exclusion chromatography (qEV), ExoEasy affinity purification (EE), Total exosome isolation kit with (TEI) and without (TEI-) protease treatment, and large vesicle sedimentation (lEVs). The image is visualized by chemiluminescence. Ladder (L) sizes are added for reference. The contrast is set automatically by the Azure 300 scanner. Arrow indicates the expected size of the CD63 protein.

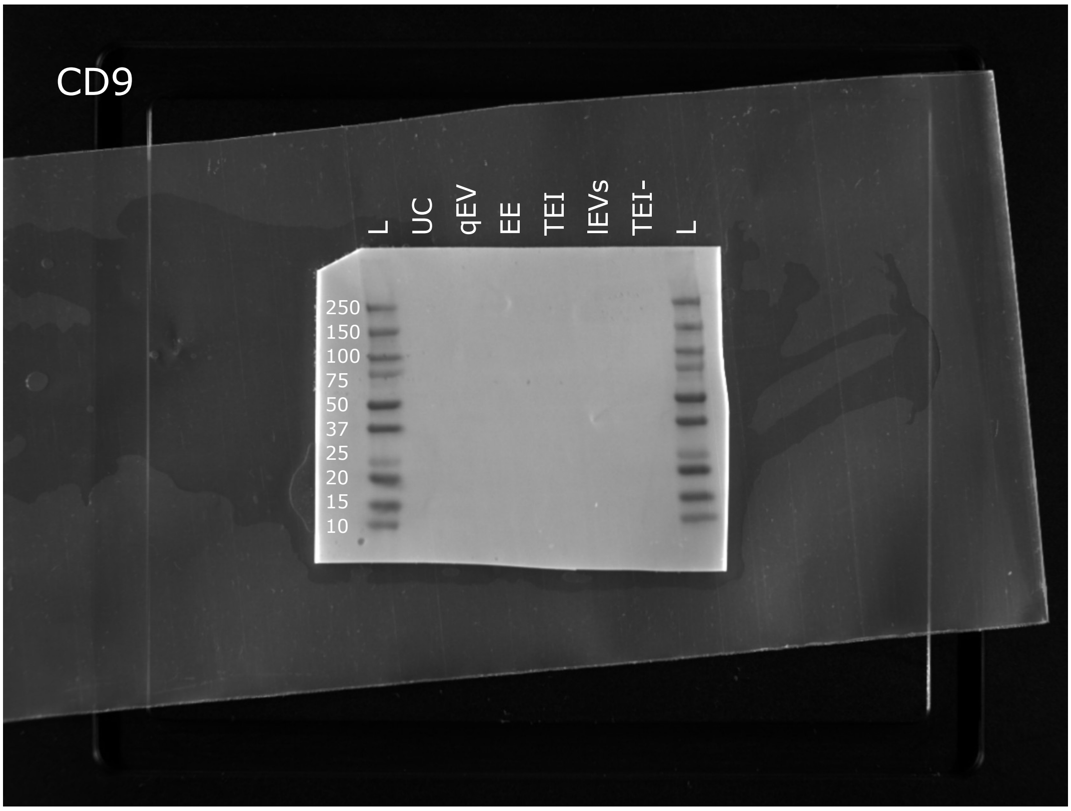

Supplemental figure S6: Uncropped, unadjusted western blot analysis of CD9 in 10 µg vesicle proteins isolated by ultracentrifugation (UC), size-exclusion chromatography (qEV), ExoEasy affinity purification (EE), Total exosome isolation kit with (TEI) and without (TEI-) protease treatment, and large vesicle sedimentation (lEVs). The image is visualized in white light. Ladder (L) sizes are added for reference.

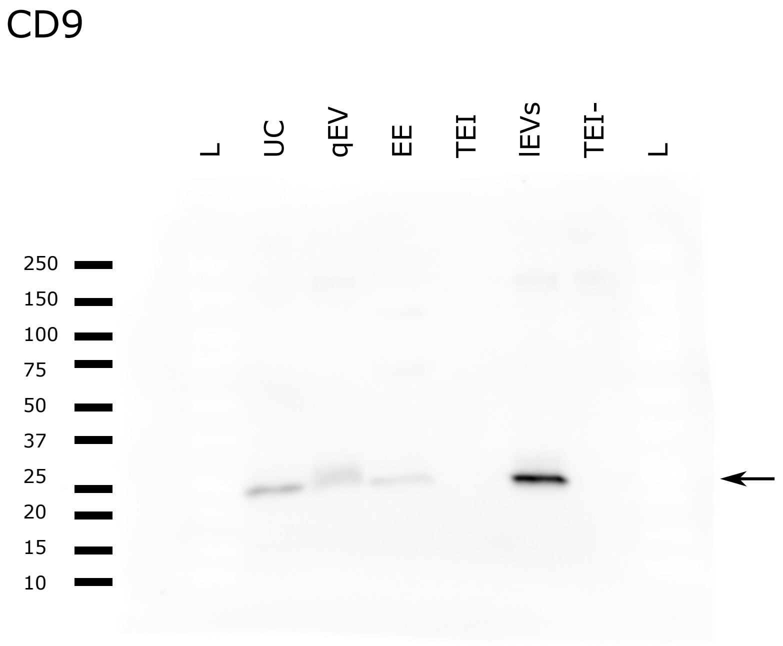

Supplemental figure S7: Uncropped, unadjusted western blot analysis of CD9 in 10 µg vesicle proteins isolated by ultracentrifugation (UC), size-exclusion chromatography (qEV), ExoEasy affinity purification (EE), Total exosome isolation kit with (TEI) and without (TEI-) protease treatment, and large vesicle sedimentation (lEVs). The image is visualized by chemiluminescence. Ladder (L) sizes are added for reference. The contrast is set automatically by the Azure 300 scanner. Arrow indicates the expected size of the CD9 protein.

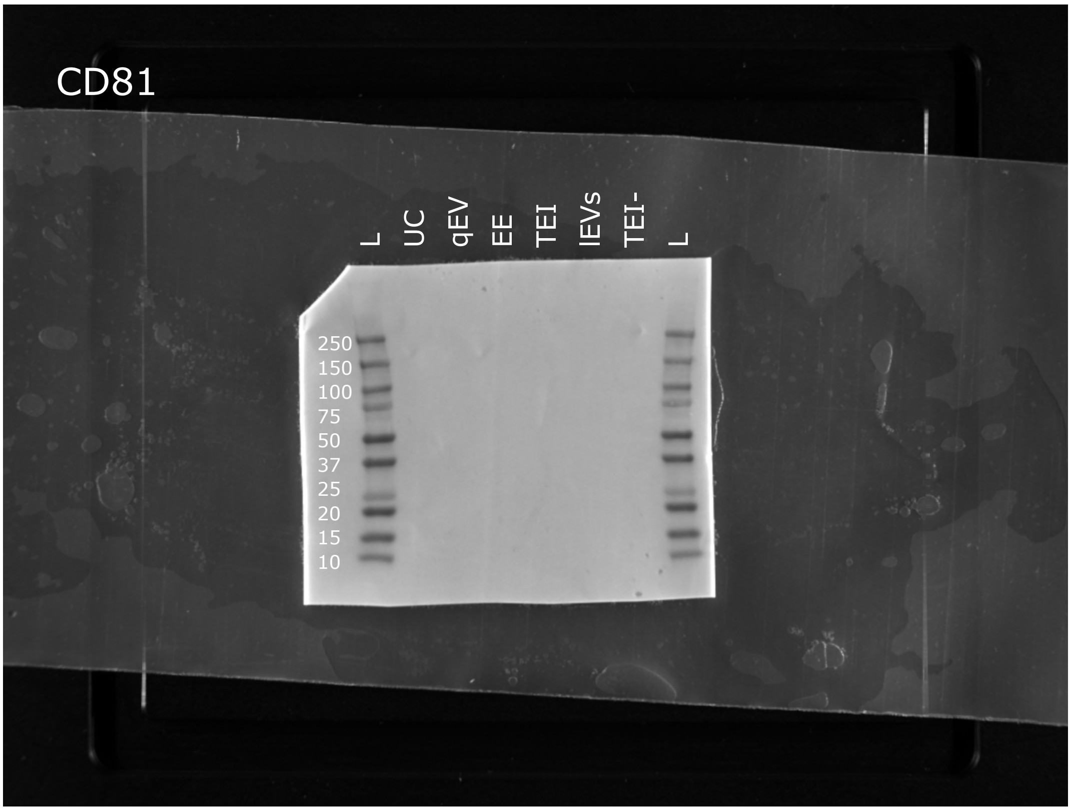

Supplemental figure S8: Uncropped, unadjusted western blot analysis of CD81 in 10 µg vesicle proteins isolated by ultracentrifugation (UC), size-exclusion chromatography (qEV), ExoEasy affinity purification (EE), Total exosome isolation kit with (TEI) and without (TEI-) protease treatment, and large vesicle sedimentation (lEVs). The image is visualized in white light. Ladder (L) sizes are added for reference.

CD81

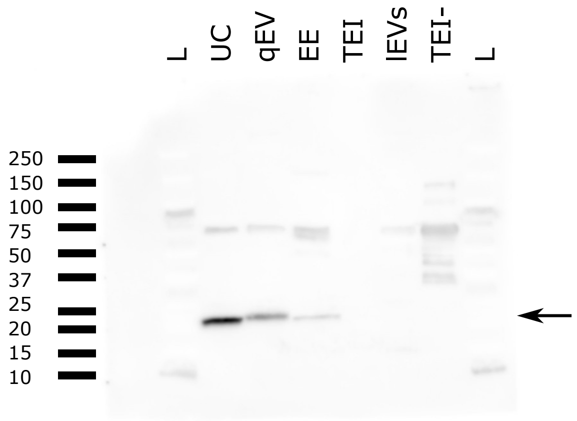

Supplemental figure S9: Uncropped, unadjusted western blot analysis of CD81 in 10 µg vesicle proteins isolated by ultracentrifugation (UC), size-exclusion chromatography (qEV), ExoEasy affinity purification (EE), Total exosome isolation kit with (TEI) and without (TEI-) protease treatment, and large vesicle sedimentation (lEVs). The image is visualized by chemiluminescence. Ladder (L) sizes are added for reference. The contrast is set automatically by the Azure 300 scanner. Arrow indicates the expected size of the CD81 protein.

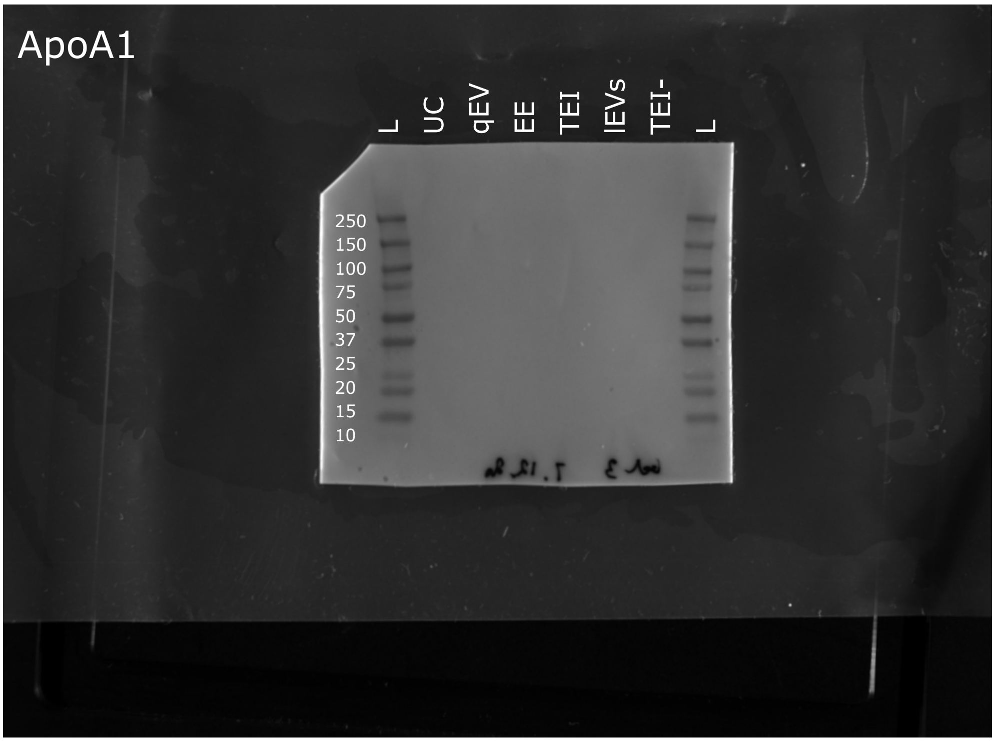

Supplemental figure S10: Uncropped, unadjusted western blot analysis of ApoA1 in 10 µg vesicle proteins isolated by ultracentrifugation (UC), size-exclusion chromatography (qEV), ExoEasy affinity purification (EE), Total exosome isolation kit with (TEI) and without (TEI-) protease treatment, and large vesicle sedimentation (lEVs). The image is visualized in white light. Ladder (L) sizes are added for reference.

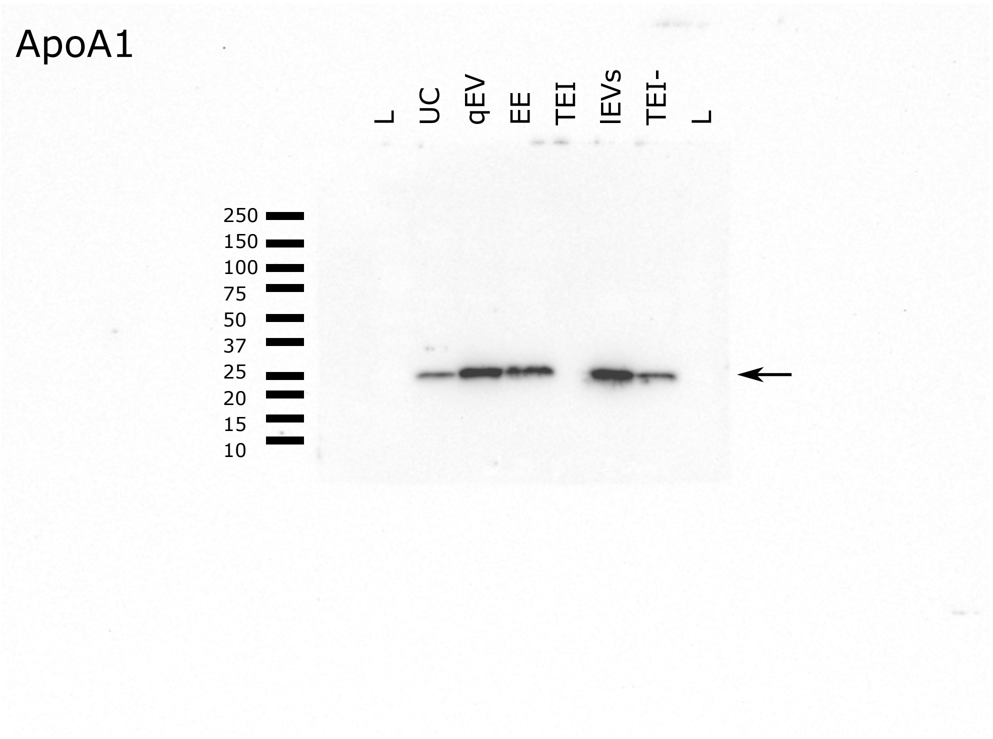

Supplemental figure S11: Uncropped, unadjusted western blot analysis of ApoA1 in 10 µg vesicle proteins isolated by ultracentrifugation (UC), size-exclusion chromatography (qEV), ExoEasy affinity purification (EE), Total exosome isolation kit with (TEI) and without (TEI-) protease treatment, and large vesicle sedimentation (lEVs). The image is visualized by chemiluminescence. The ladder (L) is not visible, size bars are added for reference. The contrast is set automatically by the Azure 300 scanner. Arrow indicates the expected size of the ApoA1 protein.
